# Effects of a wearable hand orthosis on upper and lower limb motor recovery in stroke patients: a randomized controlled trial

**DOI:** 10.3389/fbioe.2025.1600706

**Published:** 2025-05-30

**Authors:** Lijuan Xu, Jie Zhang, Qiang Liu, Yefan Cao, Nazhakaiti Aizezi, Jing Tian, Cheng Wu, Liyu Fang, Liyi Chen, Yanzheng Zhang, Xueming Pang, Yanli Lin, Jingxin Wang, Hewei Wang

**Affiliations:** ^1^ Department of Rehabilitation, Hangzhou Linping Hospital of Traditional Chinese and Western Medicine, Hangzhou, China; ^2^ Department of Occupational Rehabilitation Center, Shaanxi Provincial Rehabilitation Hospital, Shaanxi, China; ^3^ Department of Rehabilitation, Huashan Hospital, Fudan University, Shanghai, China; ^4^ Department of Bioengineering, University of Pittsburgh, Pittsburgh, PA, United States; ^5^ Department of Rehabilitation, Hangzhou Fuyang Hospital of Orthopedics of Traditional Chinese Medicine, Hangzhou, China; ^6^ Faculty of Biological Sciences, University of Leeds, Leeds, United Kingdom; ^7^ Department of Rehabilitation Medicine, Shanghai Hebin Rehabilitation Hospital, The Affiliated Hospital of Shanghai Huashan Hospital, Shanghai, China; ^8^ Department of Neurology, Hangzhou Linping Hospital of Traditional Chinese and Western Medicine, Hangzhou, China; ^9^ Department of Nursing, Hangzhou Linping Hospital of Traditional Chinese and Western Medicine, Hangzhou, China; ^10^ Department of Rehabilitation Medicine, Zhengzhou Central Hospital Affiliated to Zhengzhou University, Zhengzhou, Henan, China

**Keywords:** wearable hand orthosis, stroke, spasticity, balance, rehabilitation

## Abstract

**Objective:**

Orthoses have shown potential in addressing upper limb spasticity in stroke survivors; however, their influence on motor recovery remains controversial. This study aimed to examine the effects of a wearable hand orthosis on spasticity, motor recovery of both upper and lower limbs, balance, and activities of daily living in stroke.

**Design:**

Randomized controlled trial.

**Setting:**

Inpatient rehabilitation department.

**Participants:**

Fifty-one stroke survivors with hemiplegia were randomly assigned to either an experimental group (n = 26) or a control group (n = 25).

**Interventions:**

Both groups underwent a 4-week conventional rehabilitation program. Participants in the experimental group engaged in a self-directed training program utilizing a wearable hand orthosis for 5 h daily, whereas the control group followed the identical regimen without the use of the orthosis.

**Outcome Measures:**

Modified Ashworth Scale (MAS) for spasticity, Fugl-Meyer Assessment for upper and lower extremities (FMA-UE & FMA-LE), Berg Balance Scale (BBS) and Barthel Index (BI).

**Results:**

The experimental group showed greater improvements in FMA-UE (difference = 4.37, P = 0.022), BBS (difference = 12.37, P < 0.001), and BI (difference = 17.65, P < 0.001) compared to the control group. No significant differences were found in MAS (P = 0.654) or FMA-LE (P = 0.495). A stepwise multiple linear regression analysis revealed that improvement in FMA-UE was a significant predictor of BBS recovery in the experimental group (*r* ^2^ = 0.207, P = 0.022).

**Conclusion:**

The use of a wearable hand orthosis in self-directed training significantly improved upper limb motor recovery, balance, and ADL abilities in stroke survivors. The observed correlation between upper limb recovery and balance improvement indicates the potential of this orthosis to facilitate comprehensive rehabilitation.

## Introduction

Long-term motor dysfunction frequently follows stroke (Langhorne et al., 2009). Despite rehabilitation and intensive training (Huang et al., 2024a), 50%–60% of patients still experience motor impairments and partial dependence in ADLs (Andrenelli et al., 2015; Huang et al., 2024b). Notably, spasticity affects 20%–40% of survivors (Jung et al., 2011), limiting limb use, causing abnormal positioning, pain, contractures, and increasing fall risk (Du et al., 2024; Demetrios et al., 2013).

To address spasticity, a combination of pharmacological treatments and rehabilitation interventions has been utilized (Panahi et al., 2024; Khan et al., 2019). Stretching, commonly employed in neurological conditions (Khan et al., 2019), can be performed via self-stretching, manual assistance from therapists, or through splints/orthoses (Bovend'Eerdt et al., 2008). However, many patients require long-term rehabilitation (Khallaf et al., 2017), and conventional manual stretching is often labor-intensive and time-consuming, limiting its effectiveness.

Orthosis is an external device primarily designed to regulate abnormal joint motion and provide safe low-load force for muscle relaxation, maintenance of muscle length, and prevention of contractures (Tyson and Kent, 2011). A scoping review supported the use of static stretching with positional orthosis for the management of patients with post-stroke spasticity with grade A evidence (Suputtitada et al., 2024). Windt et al. (Windt et al., 2023) revealed that the utilization of hand braces can alleviate pain and enhance the range of daily activities, thereby improving the functional abilities of patients with hand osteoarthritis. Similarly, Tanja et al. (Oud et al., 2023) confirmed that 3D-printed orthoses can enhance daily life activities in individuals with chronic hand diseases. However, the use of orthosis for spasticity control has long been controversial, with guidelines and evidence explicitly refuting the effectiveness of hand splinting in the functional position among stroke survivors (Winstein et al., 2016; Salazar et al., 2019; Lannin and Herbert, 2003). A survey on upper extremity splinting after stroke revealed a variety of design principles, wearing schedules, splinting materials, and clinical objectives (Adrienne and Manigandan, 2011), thereby adding complexity to such controversy.

When investigating the impact of orthoses on upper limb spasticity and functional recovery, several studies have highlighted a noteworthy aspect suggesting a potential interplay between motor recovery in both the upper and lower limbs among stroke patients (Hendrickson et al., 2014). Poststroke individuals often exhibit impaired protective mechanisms for maintaining balance due to deficits in anticipatory and reactive postural adjustments involving upper limb involvement, which may increase the risk of falls (Arya et al., 2014; Wei et al., 2024). It is also reported that the spasticity of the affected elbow flexor increased as the challenge in standing balance gradually increased (Wang et al., 2020). A systematic review was conducted to examine the use of shoulder orthoses post-stroke and their potential impact on balance, gait performance, and trunk stability. The findings suggested that these orthoses may have a positive effect in improving these aspects for stroke patients (Van Bladel et al., 2020). Moreover, previous studies have demonstrated a substantial impact on the symmetrical gait pattern when employing upper limb orthoses (Van Bladel et al., 2020; Hwang and An, 2015). Additionally, wearing elastic orthoses was found to yield favorable outcomes in terms of gait and balance functions among chronic stroke patients, surpassing the benefits observed with the use of canes (Maguire et al., 2020). Taking the above evidence into account, studying the interactive effects between upper and lower limb motor recovery in stroke patients with a wearable hand orthosis is interesting and valuable. This study is particularly clinically important considering that reduced ability of balance function strongly impacts individual’s independence and quality of life (Tasseel-Ponche et al., 2022; Combs et al., 2010).

Based on previous studies, we hypothesized that: 1) wearing a hand orthosis would have an impact on upper limb spasticity and motor function recovery; 2) there would be an interaction between upper and lower limb motor recovery in stroke patients after the intervention of a wearable hand orthosis. Therefore, we designed a randomized controlled trial to investigate the long-term effects of a wearable hand orthosis on motor recovery in both upper and lower limbs. The objective was to investigate whether providing support for the hand in an antispastic position for up to 5 h daily would lead to improvements in motor recovery performance in both the upper and lower limbs.

## Methods

### Participants

This study was a randomized, sham-controlled, assessor-blinded clinical trial. Fifty-one participants were recruited through a public advertisement from the rehabilitation department of the Fifth People’s Hospital of Yuhang District between December 2018 and April 2020. All participants provided written informed consent. Ethical approval was obtained from the Ethics Committee of the Fifth People’s Hospital of Yuhang District, Hangzhou (2017LL007). The study was preregistered at the Chinese Clinical Trial Registry (No. ChiCTR1900025384). This study adhered to the principles of the Declaration of Helsinki (World medical association declaration of helsinki, 2013) and was conducted and reported in accordance with the recommendations provided by the CONSORT guideline (Schulz et al., 2010).

### Inclusion/exclusion criteria

The inclusion criteria were the following: (1) age 30–80 years; (2) first-ever cerebrovascular accident confirmed by MRI or CT as ischemic or hemorrhagic unilateral stroke; (3) onset within 1–6 months; (4) Montreal Cognitive Assessment (MoCA) score ≥20; (5) hand Brunnstrom grade I-V; (6) modified Ashworth Scale rating for wrist flexor ≤3; (7) Fugl-Meyer Assessment of Upper Extremity (FMA-UE) score ≥7, with detectable biceps and radial nerve reflexes; (8) ability to stand and walk independently or with walking aids.

The exclusion criteria were the following: (1) severe cardiac, pulmonary, hepatic, or renal dysfunction; (2) history of brain trauma, encephalitis, or other central nervous system disorders; (3) conditions significantly affecting limb sensation or movement (e.g., trauma, fractures, peripheral neuropathy); (4) balance impairment due to visual deficits, vestibular dysfunction, or vertigo; (5) botulinum toxin use within the past 3 months.

### Prior sample size estimation

We determined the necessary sample size based on a meta-analysis (Salazar et al., 2019), which revealed that static stretching was more effective than control therapy in relieving upper limb spasticity, with a mean difference of 1.8 and standard deviation of 1.6 between the two groups. To achieve a power of 0.9 and a two-tailed alpha error probability of 0.05, we estimated that a total of 38 patients would be required for this study. Accounting for an anticipated dropout rate of 15% for the Wilcoxon Rank-Sum Tests, each group required at least 23 participants.

### Randomization

Stroke survivors meeting inclusion criteria were randomized into two groups: experimental (n = 26, with wearable hand orthosis) and control (n = 25, without orthosis). Baseline assessments were conducted post-randomization. Randomization used a computer-generated number table, managed by a researcher (LYC) not involved in recruitment or interventions. Allocation sequences were concealed in sealed opaque envelopes. Participants were recruited by an independent investigator (XMP), who was not involved in randomization, intervention, or evaluation.

### Intervention

All participants received 4 weeks of in-patient intervention consisting of conventional rehabilitation and self-directed training with (experimental group) or without (control group) the wearable hand orthosis.

### Conventional rehabilitation

Patients in both groups received conventional rehabilitation at a frequency and duration of 2 h per day, 5 days per week for 4 consecutive weeks. The conventional rehabilitation encompassed physical and occupational therapies that were widely acknowledged, tailored to individual needs, and routinely practiced by experienced therapists in the rehabilitation center. This intervention targeted both upper and lower extremities with the aim of improving motor skills in activities of daily living. It incorporated techniques including muscle stretching, strengthening exercises, task-specific training, neuromuscular facilitation, etc. All interventions were conducted by experienced physical therapists who had undergone standardized training and were blinded to group assignment and outcome measurements.

### Self-directed training program

**FIGURE 1:**
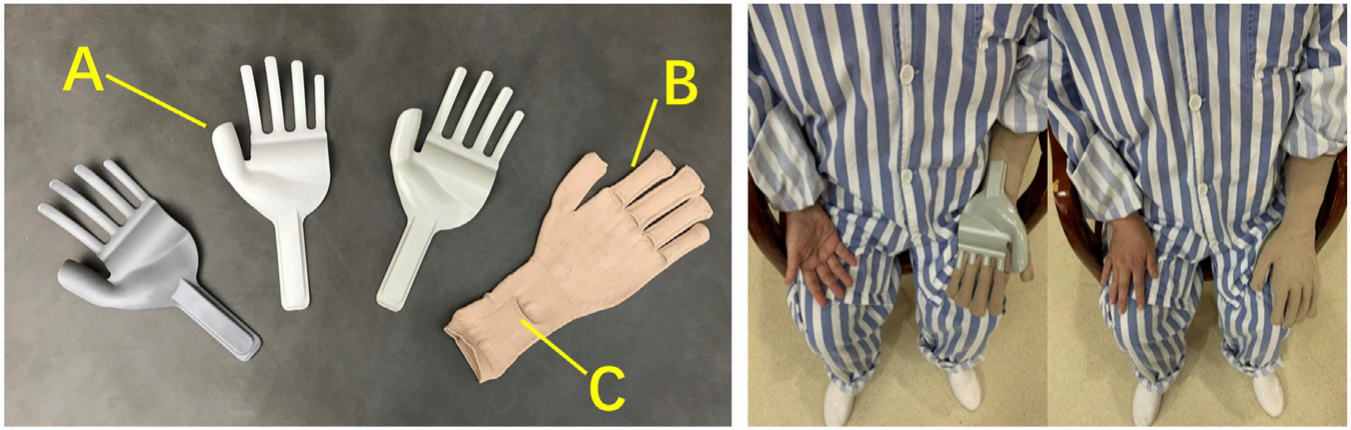
The wearable hand orthosis. **(A)** a molded plug-in for fixing the wrist and hand; **(B)** a seamless knitted glove; **(C)** a wrist strap for stabilization.

The wearable hand orthosis was designed to manage the spasticity and edema in stroke survivors based on the physiological and pathological characteristics of the impairments of hand and wrist (Figure 1). Patients in the experimental group were instructed to follow a self-directed training program with the wearable hand orthosis during rest periods (outside of rehabilitation treatment). Each wearing session should last at least 1 h, with a total daily wearing time of 5 h (Lannin and Herbert, 2003), 7 days a week, for 4 weeks. The program comprised 1 hour each of standing, walking, and upper limb joint activities, along with 2 hours of seated rest while wearing the orthosis. In contrast, the control group adhered to the same training protocols but did not utilize the orthosis. During the wearing process, blood circulation at the fingertips was monitored through small glove holes, and fingertip skin temperature was observed. Patients were instructed to remove the orthosis immediately if discomfort occurred. The skin issues like irritation or rashes, and hand symptoms such as pain, tingling, or numbness and any other adverse events (AEs) were documented.

### Outcome measures

Outcomes were assessed by an independent, trained physician (YLL) before and after 4 weeks of treatment, blinded to grouping information.

The primary outcome was assessed using the Modified Ashworth Scale (MAS) for wrist flexor spasticity (Naghdi et al., 2008). The MAS is an ordinal scale with a 6-grade criterion ranging from 0 to 4. For statistical analysis, these grades were assigned numerical scores (0, 1, 1.5, 2, 3, 4). The effectiveness of treatment depended on the change in MAS levels before and after treatment and follow-up, and the specific criteria were as follows (Li et al., 2020): (i) complete response (CR) if the original level degraded ≥2 or reduced to level 0; (ii) partial response (PR) if the original level degraded 1; and (iii) no response (NR) if the original level did not change or upgrade. The formula used was: Effective rate = total effective number/total number of cases × 100%.

Secondary outcomes included the Fugl-Meyer Assessment (FMA), Berg Balance Scale (BBS), and Barthel Index (BI). The FMA evaluates motor function in stroke patients, comprising two parts: FMA-UE (Upper Extremity) and FMA-LE (Lower Extremity). Maximum scores are 66 for FMA-UE and 34 for FMA-LE, with higher scores indicating better motor ability. It is a valid and reliable tool for evaluating stroke survivors (Gladstone et al., 2002). The BBS comprises 14 actions scored from 0 to 4, evaluating balance function. The total score is 56, with higher scores indicating better balance ability. According to a survey of 655 professionals, the BBS is widely recognized as the most commonly used assessment tool for stroke rehabilitation across various care settings (Blum and Korner-Bitensky, 2008).

The BI is a tool that assesses an individual’s ability to perform daily activities (Quinn et al., 2011). It includes 10 items: feeding, bathing, grooming, transferring, bowel and bladder control, toileting, ambulation, and stair climbing. Scores range from 0 to 100, with higher scores indicating greater independence in daily functioning.

### Statistical analysis

An independent statistician (JZ) conducted the statistical analysis blinded to the interventions. SPSS (Version 24.0, IBM, Chicago, IL) was used for all analyses. Continuous data are presented as mean ± SD, and categorical variables as numbers (%).

Baseline differences between the characteristics of patients in the experimental and control groups were compared using the t-test, Wilcoxon rank-sum test, chi-square test, or Fisher’s exact test (Table 1). Data normality was assessed with the Shapiro-Wilk test. Changes in the clinical outcome measure scores after training were analyzed using the t-test (FMA-UE, FMA-LE, BBS and BI) or Wilcoxon rank-sum test (MAS). Measurement data were expressed as mean ± standard deviation if normally distributed, and median (interquartile range) if not normally distributed. Count and rank data are presented as total numbers. The statistical significance of all other tests was set at p < 0.05.

**TABLE 1:** Demographic information at baseline by group.

Patient characteristic	Experiment group n = 25	Control group n = 23	P value	t/χ2
Age, years	57.36 ± 11.11	62.48 ± 7.15	0.066	1.880^a^
Gender			0.807	0.060^b^
Male (n (%))	15 (60.0)	13 (56.5)		
Female (n (%))	10 (40.0)	10 (43.5)		
Affected side			0.971	0.001^b^
Left (n (%))	14 (56.0)	13 (56.5)		
Right (n (%))	11 (44.0)	10 (43.5)		
Onset time, months	2.44 ± 1.56	2.57 ± 1.97	0.807	0.245^a^
Stroke type			0.852	0.035^b^
Infarction	19 (76.0)	18 (78.3)		
Hemorrhage	6 (24.0)	5 (21.7)		
Fugl-Meyer Assessment				
Upper Extremity	16.44 ± 11.72	16.22 ± 11.66	0.948	−0.066^a^
Hand + Wrist	8.44 ± 9.24	6.65 ± 8.54	0.491	−0.694^a^
Lower Extremity	18.80 ± 6.34	17.70 ± 7.36	0.579	−0.558^a^
Total	43.68 ± 23.22	40.57 ± 24.65	0.654	−0.451^a^
Barthel Index	59.20 ± 17.18	61.96 ± 21.94	0.629	0.487^a^
Berg Balance Scale	21.84 ± 14.38	24.52 ± 18.90	0.581	0.556^a^
Modified Ashworth Scale			0.350	0.935^c^
0	13 (52.0)	7 (30.4)		
1	4 (16.0)	9 (39.1)		
1+	5 (20.0)	4 (17.4)		
2	3 (12.0)	2 (8.7)		
3	0 (0.0)	1 (4.3)		

Data are presented as the mean ± standard deviation or numbers (%).

^a^ Independent-sample t test.

^b^ Pearson’s chi-square test.

^c^ Wilcoxon rank-sum test.

To identify the factors contributing to superior recovery of the BBS in the experimental group and to examine the interactive effects between upper and lower limb motor recovery, stepwise multiple linear regression analysis was employed to evaluate the predictive value of relevant indicators on BBS improvement within each group. Multicollinearity was evaluated using collinearity diagnostics. Statistical significance was set at P < 0.05. A per-protocol analysis was conducted, including only participants who completed all assessments (Schoenfeld, 2005).

## Results

### Recruitment and retention

**FIGURE 2:**
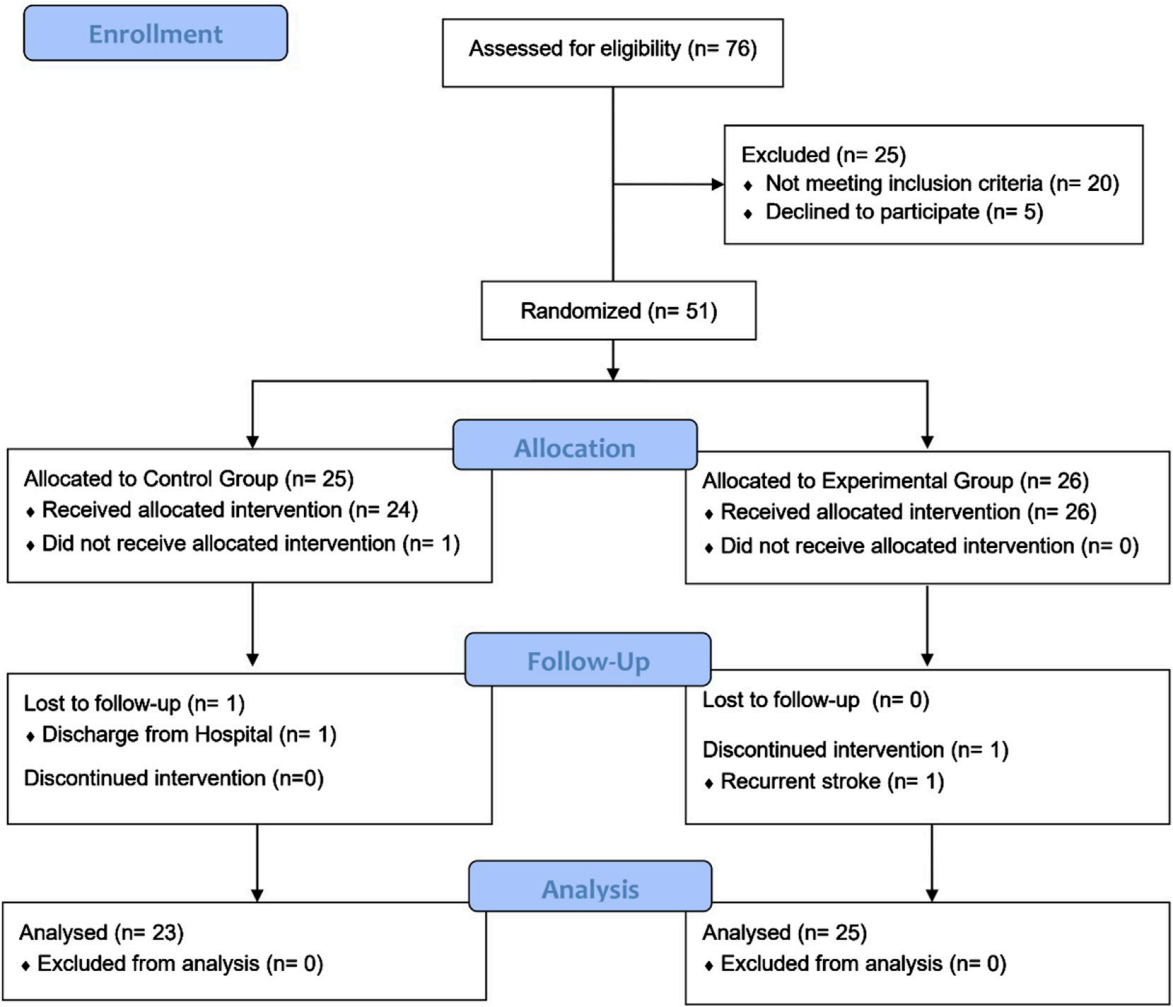
CONSORT flow diagram.

From an initial pool of 76 stroke survivors, 56 met the study criteria, and 51 consented to participate. No significant differences (P > 0.05) were observed between groups in baseline demographics, motor abilities of upper and lower limbs, or activities of daily living (Table 1). Figure 2 provides a flow diagram detailing participant recruitment, allocation, and follow-up.

### Primary outcome

By the end of treatment, the MAS score for the affected wrist flexor decreased in both the experimental group (effective rate = 40.0%) and the control group (effective rate = 39.1%). In the experimental group, 28.0% of participants achieved CR, 12.0% achieved PR, and 60.0% showed NR. In comparison, in the control group, 13.0% of patients achieved CR, 26.1% achieved PR, and 60.9% showed NR. The between-group difference of MAS change before and after treatment was not statistically significant (P = 0.068) (Table 2).

**TABLE 2:** Comparison of change of MAS.

Group	4 weeks Post intervention–Pre intervention, n (%)					
	CR	PR	NR	Total	Effective rate (%)	P/Z
Experimental group	7 (28.0)	3 (12.0)	15 (60.0)	25	40.0%	0.654/0.449
Control group	3 (13.0)	6 (26.1)	14 (60.9)	23	39.1%	
Total	10 (20.8)	9 (18.8)	29 (60.4)	48	39.6%	

T0, baseline test; T1, test after 4 weeks; CR, complete response, if the original level degraded ≥2 or reduced to level of 0; PR, partial response, if the original level degraded 1; NR, no response, if the original level did not change or upgrade.

### Secondary outcomes

At 4 weeks, both groups exhibited significant improvements in FMA-UE, FMA-LE, BBS, and BI compared to the baseline (Table 3). Significant between-group differences were found in FMA-UE (difference: 4.37 points, 95% CI: 0.67 to 8.07, P = 0.022), BBS (difference: 12.37 points, 95% CI: 5.84 to 18.90, P < 0.001), and BI (difference: 17.65 points, 95% CI: 10.37 to 24.94, P < 0.001). However, there were no significant between-group differences in FMA-LE (difference: 1.14 points, 95% CI: -2.20 to 4.49, P = 0.495) (Table 4).

**TABLE 3:** Changes in Secondary outcomes in both groups.

Outcomes	Experimental group (n = 25)			Control group (n = 23)		
	Baseline	After 4 weeks	P/t value	Baseline	After 4 weeks	P/t value
FMA-UE	16.44 ± 11.72	25.20 ± 14.03	<0.001/5.435	16.22 ± 11.66	20.61 ± 12.10	<0.001/5.633
FMA-LE	18.80 ± 6.34	25.16 ± 472	<0.001/5.120	17.70 ± 7.36	22.91 ± 5.91	<0.001/4.816
BBS	21.84 ± 14.38	40.56 ± 11.42	<0.001/6.378	24.52 ± 18.90	30.87 ± 16.57	<0.001/5.674
BI	59.20 ± 17.18	86.20 ± 14.24	<0.001/8.332	61.96 ± 21.94	71.30 ± 17.85	<0.001/6.951

**TABLE 4:** Comparison of treatment effects between two groups in outcomes.

Outcomes	Change scores (After 4 weeks - Baseline)		Difference between groups (95% CI)	P/t value
	Experimental group (n = 25)	Control group (n = 23)		
FMA-UE	8.76 ± 8.06	4.39 ± 3.74	4.37 (0.67–8.07)	0.022/2.374
FMA-LE	6.36 ± 6.21	5.22 ± 5.20	1.14 (−2.20–4.49)	0.495/0.688
BBS	18.72 ± 14.68	6.35 ± 5.37	12.37 (5.84–18.90)	<0.001/3.813
BI	27.00 ± 16.20	9.35 ± 6.45	17.65 (10.37–24.94)	<0.001/4.878

### Treatment fidelity and adverse events

Interventions with or without wearable hand orthosis were not associated with serious adverse events. Occasional mild skin redness and sweaty palms were reported by some patients; however, no pain or dermal damage occurred. Symptoms resolved within minutes after removing the orthosis. Treatment compliance was excellent, with 50 (98.04%) participants completing their assigned interventions and 48 (94.12%) receiving all follow-up assessments. Reasons for withdrawal are detailed in Figure 2.

### Relationship between BBS and upper limb motor function

**FIGURE 3:**
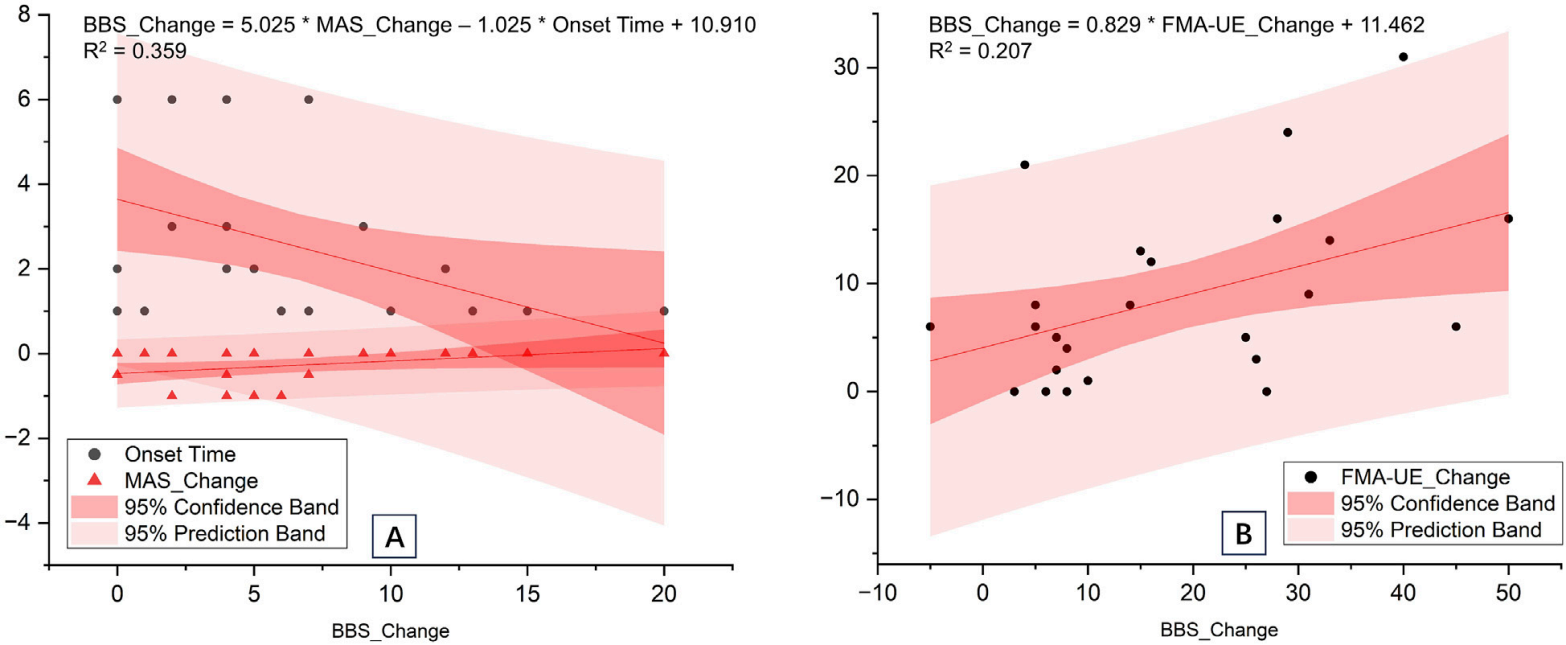
The optimal models for predicting balance recovery using multiple linear regression analysis. **(A)** Control group; **(B)** Experimental group.

Upper limb motor function indicators (FMA-UE, FMA-hand, MAS) and key baseline characteristics (onset time, age) were analyzed using stepwise multiple linear regression. Table 5 shows three models generated for the two groups. The optimal models for predicting the BBS in the experimental group were based on a single indicator (BBS_Change = 0.829 * FMA-UE_Change +11.46, r^2^ = 0.207), whereas in the control group, they relied on two indicators (BBS_Change = 5.025 * MAS_Change – 1.025 * Onset Time +10.910, r^2^ = 0.359) (Figure 3).

**TABLE 5:** The results of multiple linear regression coefficients in each group.

Group	Model	Predictors	Unstandardized Coefficients		Standardized Coefficients	*t*	*P*	r^2^	Collinearity Statistics	
			*b*	*SE*	*β*				Tolerance	VIF
Experimental Group	1	(Constant)	11.462	3.988		2.874	0.009	0.207		
		FMA-UE Change	0.829	0.338	0.455	2.450	0.022		1.000	1.000
Control Group	1	(Constant)	9.568	1.689		5.663	<0.001	0.213		
		Onset time (months)	−1.255	0.526	−0.462	−2.385	0.027		1.000	1.000
	2	(Constant)	10.910	1.685		6.476	<0.001	0.359		
		Onset time (months)	−1.205	0.487	−0.443	−2.473	0.023		0.998	1.002
		MAS Change	5.205	2.442	0.382	2.132	0.046		0.998	1.002

Dependent Variable: BBS Change; VIF: Variance Inflation Factor.

## Discussion

The study aimed to investigate the effects of wearing a hand orthosis providing antispastic support for 4 weeks, during which patients engaged in self-directed training programs for up to 5 h per day. Results showed no significant reduction in wrist flexion spasticity in the experimental group compared to controls. However, the experimental group demonstrated superior recovery in FMA-UE, BBS, and BI scores, while no significant improvement was observed in FMA-LE scores. Multiple linear regression analysis showed that FMA-UE_Change was the sole predictor of BBS_Change in the experimental group. In the control group, MAS_Change and Onset Time predicted BBS_Change. Overall, participants demonstrated high tolerance and excellent compliance with the orthosis.

Despite uncertainty regarding optimal treatment parameters, orthosis remains a preferred adjunctive treatment for post-stroke spasticity (Hesse et al., 2013). Orthoses help stretch affected muscles, control abnormal joint motion, improve stability and alignment, and enhance mobility and functionality (Jiang et al., 2021). Kerr et al. (2020) showed significant improvements in hand function and upper limb activities using static splinting, supported by moderate evidence. In our study, the experimental group exhibited a more pronounced reduction in MAS compared to the control group; however, this difference did not reach statistical significance. Therefore, the use of orthoses for preventing spasticity remains a topic of ongoing debate (Winstein et al., 2016). A randomized controlled trial investigating volar and dorsal splinting effects on wrist flexor muscle spasticity in stroke patients found effects on MAS score or passive range of motion (Basaran et al., 2012). Several recent reviews highlight uncertainties in the mechanisms of spastic muscle stretching and present conflicting evidence regarding its effectiveness and potential injury risks (Salazar et al., 2019; Mehraban Jahromi et al., 2024). However, Consistent with prior studies (Salazar et al., 2019; Lannin and Herbert, 2003; Lannin et al., 2007), participants in our study were able to tolerate wearing orthosis for several hours a day continuously for weeks without serious adverse events. This prompted consideration of potential benefits in areas beyond spasticity control.

The observed differences in FMA-UE scores between groups suggest that incorporating hand orthoses into self-directed training programs enhanced upper limb motor recovery. Currently, few studies report on the effectiveness of hand orthoses for post-stroke motor function recovery (Wong et al., 2023). A recent systematic review found a positive effect on upper limb activity, though based on two small studies (n < 30) (Alexander et al., 2022). Wong et al. (Wong et al., 2023) evaluated a dynamic hand orthosis for aiding upper limb recovery in stroke patients. The device maintained optimal wrist and hand positioning during grasp training, resulting in improved upper limb function as assessed by the action research arm test. However, one moderate-quality study found no difference in wrist and hand motor function after using an inflatable pressure splint that positioned the shoulder at 90° flexion and maximum external rotation with full elbow extension for 30 min daily (Poole et al., 1990). The disparity may be due to two factors: firstly, this study did not include active upper limb training while wearing the orthosis; secondly, patients only received 30 min of orthosis intervention per day, which was much less than in other studies. The disparity may be due to two factors: first, this study did not include active upper limb training with the orthosis; second, patients received only 30 min of orthosis intervention daily, significantly less than in other studies.

Our study found that the experimental group showed greater improvements in BBS scores than the control group. Given that the only difference between the groups was the use of hand orthosis in self-directed training, we suggest that incorporating hand orthosis into standing and walking training can enhance balance recovery. The latest research findings have also indicated that intervention targeting the upper limb holds the potential to enhance lower limb motor function in individuals with hemiplegia (Dash et al., 2020; Kim et al., 2017; Park and Oh, 2020). Kim et al. demonstrated that shoulder stabilization training in a standing position can effectively enhance hand function and walking ability in patients with hemiplegia (Kim et al., 2017). Park et al. (Park and Oh, 2020) found that shoulder immobilization devices improved walking speed, stride length, and support phase duration in stroke patients compared to the control group, suggesting their potential to enhance gait efficiency. The study by Khumsapsiri et al. (Khumsapsiri et al., 2018) demonstrated that a multi-directional arm extension orthosis enhanced knee loading and proprioception, thereby enhancing balance function in stroke patients.

This phenomenon can be described as the interaction between upper and lower limb motor recovery, facilitated by the use of a hand orthosis. Our regression analysis revealed a moderate correlation between balance recovery, time since stroke onset, and reduced spasticity in the control group. Prior research has indicated that the development of upper limb spasticity may be associated with balance recovery in stroke patients (Bakheit and Sawyer, 2002). Additionally, botulinum toxin injections in the spastic upper extremities of hemiplegic patients have been shown to improve postural balance and gait function (Lee et al., 2023). In contrast, within the experimental group, the orthosis altered the effects of spasticity, diverging from typical outcomes. Specifically, the moderate correlation between BBS_Change and FMA-UE_Change indicated that improvements in upper limb function contributed significantly to balance recovery. Another possible explanation is that the orthosis might enhance balance by correcting abnormal upper limb motor patterns and compensatory strategies commonly employed by stroke patients during walking (Jiang et al., 2021; Kahn et al., 2021).

In this study, there was no statistically significant difference in the FMA-LE scores between the two groups before and after treatment. This suggests that improvements in motor ability are typically seen only in functions directly targeted by rehabilitation (Shahid et al., 2023). Van et al. proposed that lower limb motor control could only be improved through isolated lower limb movements, gait training, intensive weight shifting, and a limited reliance on upper limb assistive devices (van Elk et al., 2013). Another reason for this result may be related to the “ceiling effect” of FMA-LE (Thrane et al., 2019). Given that most patients included in the study had good baseline walking ability, the assessment tool may not be sensitive enough to detect differences in lower limb motor enhancement between the two groups (Zhang et al., 2024).

The current study had several limitations that warrant consideration. First, the lack of long-term follow-up data restricted the ability to assess sustained outcomes. Second, there was limited oversight of patients' self-directed training, which may have influenced the results. Third, while shoulder and hand pain could potentially affect the development of spasticity, this factor was not incorporated into the predictive analysis in the present study. Lastly, this study did not incorporate neuroimaging or electrophysiological tools to investigate the underlying mechanisms of the intervention’s efficacy (Yu et al., 2024). Given that this was a preliminary investigation, these limitations were anticipated. Future studies will focus on addressing these issues and further enhancing our understanding of the mechanism underlying the interaction between upper and lower limb motor recovery through the application of a hand orthosis.

## Conclusion

The wearable hand orthosis, used with self-directed training for 5 h daily over 4 weeks, did not significantly reduce wrist flexion spasticity compared to control therapy. However, it improved upper limb motor function, balance, and independence in daily activities more effectively. Our study also found a significant correlation between enhanced upper limb function and balance recovery after using the orthosis. Further research is needed to explore the neural mechanisms behind this recovery pattern.

## Data Availability

The raw data supporting the conclusions of this article will be made available by the authors, without undue reservation.
