# Supplementary material for: Effects of a wearable hand orthosis on upper and lower limb motor recovery in stroke patients: a randomized controlled trial
Source: Front Bioeng Biotechnol. 2025 May 30;13:1600706. doi: 10.3389/fbioe.2025.1600706 (PMC12162502; doi:10.3389/fbioe.2025.1600706)
Supplement: Supplementary file 1 [file Table1.docx]

Supplement Material Content

[S1. The design of the wearable hand orthosis 2](#_Toc197980346)

[S2. Figure - Correlation matrix of clinical and functional outcome measures of controlled group. 3](#_Toc197980347)

[S3. Figure - Correlation matrix of clinical and functional outcome measures of experimental group. 4](#_Toc197980348)

**S1. The design of the wearable hand orthosis**

The wearable hand orthosis consisted of three parts: a molded plug-in for fixing the wrist and hand, a seamless knitted glove, and a wrist strap for stabilization. The plug-in, made of rigid synthetic material, supported the paralyzed side hand and wrist in a neutral position (Figure1.A). When wearing the hand orthosis, the patient's four fingers were appropriately separated, with slight abduction of the thumb. This posture aimed to control spasticity in the flexor muscles of the wrist and fingers. This seamless knitted glove was made using a one-piece molding technique and was produced with polyester/ammonia coated yarn (Figure1.B). It was tightly woven to avoid the compression of side seams that could impede blood circulation, while ensuring a comfortable fit with continuous pressure. Additionally, the glove provided moderate and even resting pressure to promote lymphatic drainage in the hands and provide sensory input. Considering the different sizes of patients' palms, the gloves came in three different sizes: S, M, and L. When wearing gloves, patients were guided and instructed by specialized therapists (QL) to sequentially wear gloves on the affected side's wrist and hand, install attachments, and finally secure straps (Figure1.C).


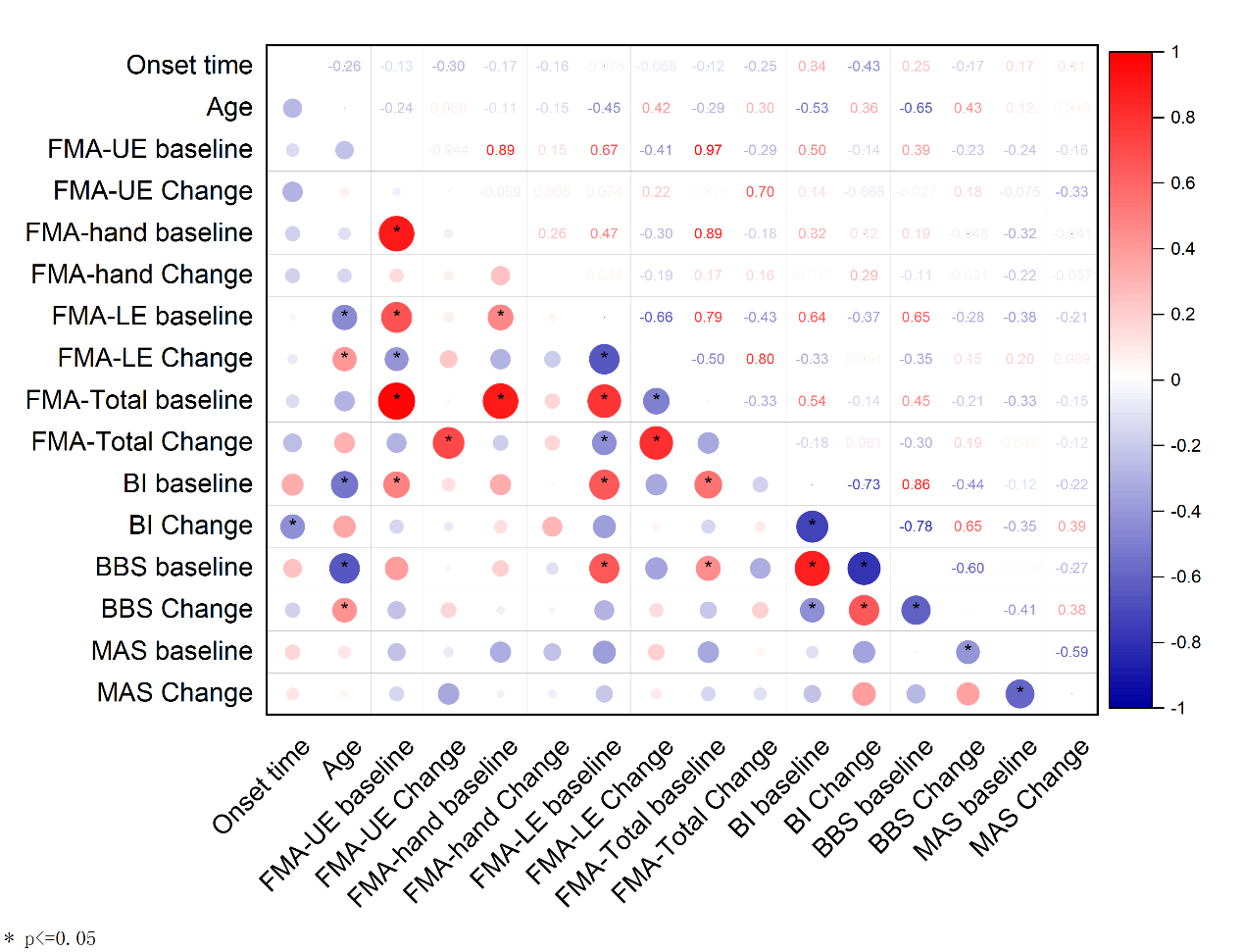


**S2. Figure - Correlation matrix of clinical and functional outcome measures of controlled group.**


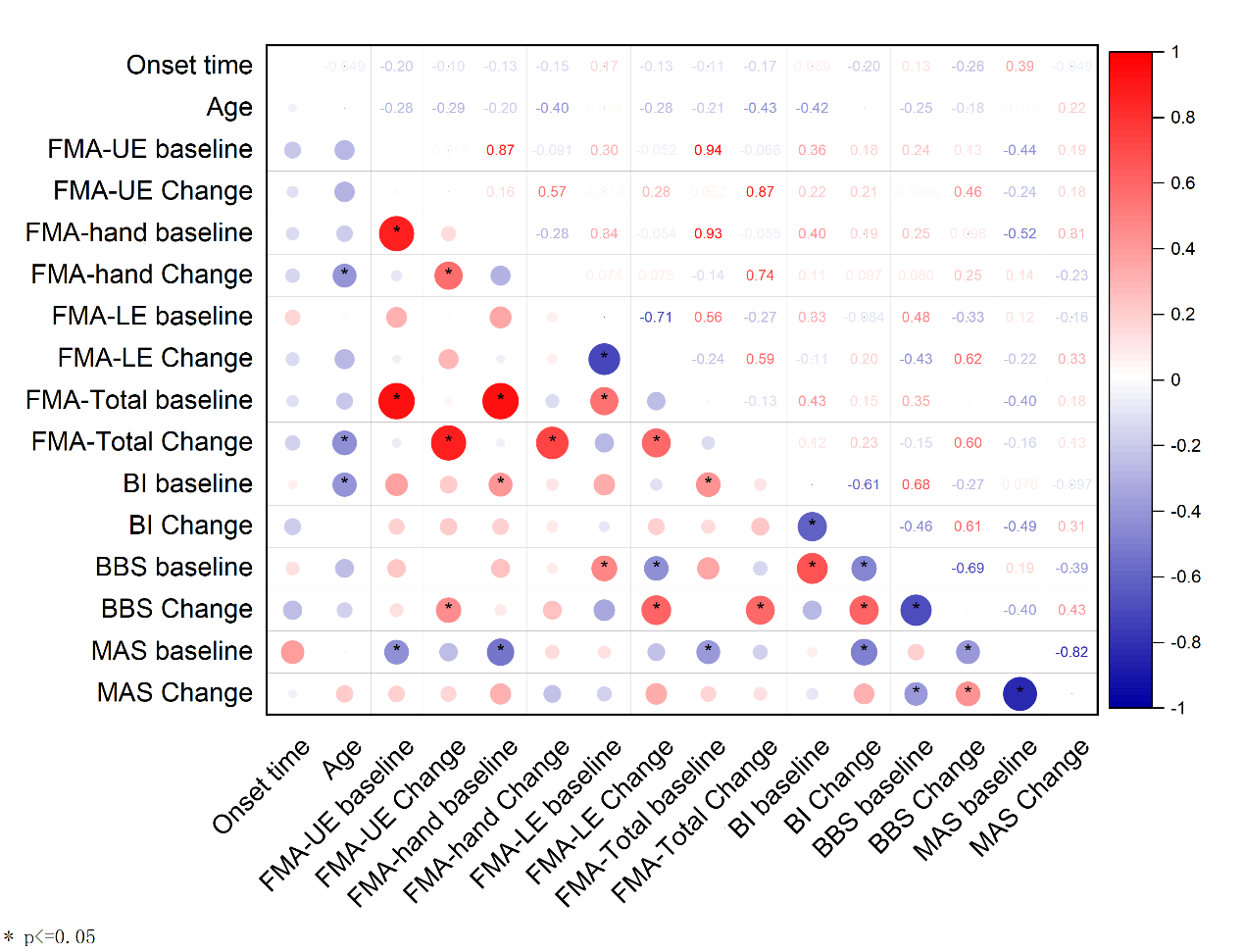


**S3. Figure - Correlation matrix of clinical and functional outcome measures of experimental group.**
